# Oral Microbiota in Children with Cleft Lip and Palate: A Systematic Review

**DOI:** 10.3390/jcm12185867

**Published:** 2023-09-09

**Authors:** Jacek Świtała, Magdalena Sycińska-Dziarnowska, Gianrico Spagnuolo, Krzysztof Woźniak, Katarzyna Mańkowska, Liliana Szyszka-Sommerfeld

**Affiliations:** 1 Department of Orthodontics, Pomeranian Medical University in Szczecin, Al. Powst. Wlkp. 72, 70111 Szczecin, Poland; magdadziarnowska@gmail.com (M.S.-D.); krzysztof.wozniak@pum.edu.pl (K.W.); liliana.szyszka.sommerfeld@pum.edu.pl (L.S.-S.); 2 Department of Neurosciences, Reproductive and Odontostomatological Sciences, University of Naples “Federico II”, 80131 Napoli, Italy; gspagnuo@unina.it; 3 School of Dentistry, College of Dental Medicine, Kaohsiung Medical University, Kaohsiung 80708, Taiwan; 4 Department of Laboratory Medicine, Pomeranian Medical University, Al. Powst. Wlkp. 72, 70111 Szczecin, Poland; katarzyna.mankowska@pum.edu.pl

**Keywords:** cleft lip and palate, congenital facial deformity, dental caries, oral health, oral microbiota, orthodontic treatment

## Abstract

Background: Cleft in the lip and/or palate (CLP) is a congenital facial deformity that significantly impacts the oral cavity’s structure and function. This malformation can affect the oral microbiota. The objective of this systematic review was to examine and consolidate the current scientific evidence on the oral microflora in children with CLP. Methods: The search strategy included the PubMed, PubMed Central, Web of Science, Scopus, and Embase databases. The inclusion criteria were studies assessing oral microbiota in children with CLP. The Newcastle–Ottawa Scale (NOS) was used to evaluate the quality of the included studies. Results: The search strategy identified 422 potential articles. Twelve papers met the inclusion criteria. High heterogeneity was observed in methodologies, sample sites, and patient characteristics. Eight studies assessed the levels of *Streptococcus mutans* and *Lactobacillus* in saliva, with some reporting significantly higher levels in the cleft group compared to controls, while others found no differences. One study reported a significantly higher colonization rate of *Candida* species in patients with cleft lip and/or palate. Conclusion: The results of the available studies are unclear. Further research is needed to gain a comprehensive understanding of the oral microbiota and potential implications for oral health management in this population. The review was not registered Registration Statement.

## 1. Introduction

A cleft in the lip and/or palate (CLP) is the most common congenital facial deformity that significantly affects the structure and function of the oral cavity, resulting in modifications to one’s facial features [1,2,3,4]. Individuals with CLP may have serious functional problems with sucking, swallowing, chewing, speaking, breathing, and social integration, and require comprehensive and long-term rehabilitation beginning in infancy and throughout adulthood [5,6,7]. Orthodontic treatment plays a crucial role in the multidisciplinary care of these patients [8,9]. However, it is important to recognize that both the presence of malocclusion and the use of orthodontic appliances can modify the local oral environment, affecting the condition of periodontal tissues, dental hard tissues, composition and features of saliva, as well as oral microbiota [4,10,11,12].

Moreover, orthodontic appliances contribute to increased plaque accumulation and the quantity of exfoliated epithelial cells around the brackets and may hinder effective self-cleaning of teeth, thus impacting the condition of gingival tissues and dental structures [12]. Increased dental plaque accumulation may contribute to changes in the species composition and number of bacteria detected [7,13]. Consequently, individuals with CLP may face an elevated risk of infectious oral diseases, such as caries and periodontal problems/diseases [12]. The presence of orthodontic appliances can promote the colonization of cariogenic bacteria, such as *Streptococcus mutans* (SM) and the late colonizers *Lactobacillus* (LB), exacerbating the susceptibility to dental caries in CLP patients undergoing orthodontic treatment [14]. In patients with CLP, apart from the risk related to use of the orthodontic appliances, there are additional factors responsible for poor oral health and difficulties in maintaining adequate plaque control. These include the cleft deformity and anatomical changes in the maxillary segments, as well as a common fear of teeth brushing in the cleft area. Also significant is the loss of lip elasticity around the cleft scar making access to the oral cavity difficult, drying of the mucosa and teeth in the cleft area due to lip insufficiency, and prolonged oral cleansing time favoring the growth of acidophilic bacteria [12,14,15].

In the available literature, there are few data on the assessment of the oral microbiota in patients with congenital facial defects, such as cleft lip and/or palate. In addition, existing papers differ in terms of, for example, study groups and outcome measures [4,7,14,15,16,17]. Therefore, the primary objective of this systematic review was to meticulously examine and consolidate the current scientific evidence pertaining to the oral microflora in children affected by cleft lip and/or palate. The consolidation of this scientific evidence will not only summarize the existing scientific evidence but also offer valuable insights into the microbial composition of the oral cavity in this population. This synthesis of information will play a key role in advancing our understanding of microbial dynamics and potential implications for oral health management in children with CLP.

## 2. Materials and Methods

### 2.1. Search Strategy

This systematic review was conducted in accordance with the “Preferred Reporting Items for Systematic Reviews and Meta-Analyses” (PRISMA) (Supplementary Tables S1 and S2) [18].

In accordance with PICO [19], the framework of the present systematic review is as follows: Population (P): children with CLP; Intervention (I): analysis of oral microbiota composition and/or oral microbiome with a concomitant clinical oral examination. No restriction was applied for the type of microbiological technique used to sample and analyze the oral microbiota/microbiome; Comparison (C): cleft children versus non-cleft, healthy children; Outcomes (O): oral microbiota composition. The PICO question was as follows: “Does oral microbiota composition of children with cleft lip and/or palate differ from the oral microbiota of the healthy population without cleft?” A search of the following databases was performed: PubMed, Pubmed Central, Web of Science, Scopus, Embase using the following keywords: “cleft lip and palate” AND “children” AND (“oral microbiology” OR “oral microbiota” OR “oral microbiome” OR “oral microflora”).

Two independent reviewers (J.Ś. and L.S.-S.), conducted an exhaustive literature search without any limitations on publication dates. All relevant publications were examined, regardless of the language, in an unbiased manner. The final search was completed on 31 May 2023, ensuring that all available literature was considered.

### 2.2. Eligibility Criteria

The following inclusion criteria were applied for this systematic review:

- Study type: all types of observational studies on oral microbiota of patients with CLP,
- Outcome of interest: oral microbiota composition (quality and quantity of bacterial species and pathogens related to oral diseases),
- Object of the study: microbiological analysis of oral microbiota; comparison of oral microbiota composition between the CLP and control groups,
- Participants: human patients between 3 and 18 years old; children with primary, mixed, or permanent dentition.

CLP group: non-syndromic children with cleft lip and/or palate

Control group: healthy children with no CLP

The exclusion criteria were as follows: ineligible study design; ineligible outcome measure, e.g., studies concerning the influence of cleft surgery on the oral microbiota; ineligible population, e.g., studies on adult and/or syndromic CLP patients, studies on newborns or children under 3 years; case reports, reviews, and animal studies.

### 2.3. Data Extraction

Following the removal of duplicate publications, the titles and abstracts of the remaining studies were carefully reviewed by the first author (J.Ś.), then assessed by the second author (L.S.-S.) to identify potentially eligible studies. Subsequently, the full texts of the selected papers were thoroughly examined, and based on pre-established inclusion and exclusion criteria, they were either included or excluded. Only studies that compared the oral microbiota in children with clefts to those without clefts were considered for inclusion. Any uncertainties or ambiguities encountered during the process were resolved through discussions between the two authors. To assess the level of agreement between the authors, the Cohen’s Kappa statistic was employed. During the review process, relevant information pertaining to various aspects of the included studies, such as study design, participant characteristics, outcome measures (including measurement tools, procedures, and data analysis), as well as the principal findings, were gathered. A reviewer (J.Ś.) collected the results from each study and documented them in an Excel spreadsheet.

### 2.4. Quality Assessment

The Newcastle–Ottawa Scale (NOS) was used to evaluate the quality of the studies included in the review [20]. The quality assessment of the selected studies was performed according to the star score of the NOS, based on which * are assigned to three criteria, i.e., selection (with a maximum of 4 stars [****]), comparability (with a maximum of 2 stars [**]), and outcome (with a maximum of 3 stars [***]) for a maximum of 9 stars. Higher scores indicate lower risk of bias. The quality assessment was performed independently by two authors (J.Ś. and L.S.-S.). All ambiguities were resolved through discussions between the reviewers. Cohen’s Kappa coefficient for agreement between the authors was calculated.

## 3. Results

**Figure 1:**
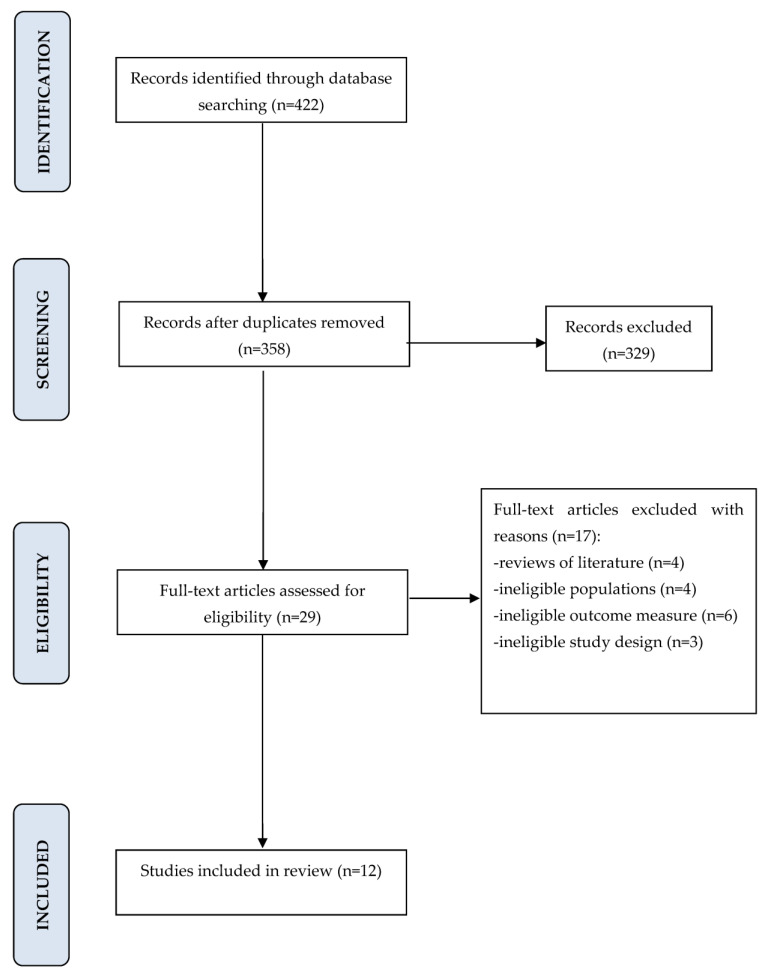
PRISMA flow diagram for the search strategy.

Using the search strategy described, 422 potential articles were identified: 43 from PubMed, 303 from PubMed Central, 46 from Embase, 19 from Scopus, and 11 from Web of science. After removing of 64 duplicates, 358 articles were analyzed. After carefully examining the titles and abstracts, a total of 329 papers were excluded from the review based on the predetermined inclusion and exclusion criteria. Out of the remaining 29 articles, 17 were further excluded due to being literature reviews, studies with ineligible study designs or outcome measures, or involving ineligible populations. Ultimately, a total of 12 papers were deemed suitable for inclusion in the review. To visualize the entire process, a Prisma Flow Diagram (Figure 1, Flow diagram) was created. The agreement between the two reviewers was assessed using the Kappa statistic, yielding a high value of 0.96, indicating strong consensus. Table 1 and Table 2 present the key characteristics of each study that was included in the review.

The quality assessment results for each study are summarized in Table 3. The agreement between the authors was evaluated using the Cohen’s Kappa coefficient, resulting in a robust value of 0.93. Based on the NOS assessment [20], most of the studies had a score of 6/9 [7,14,16,21,22,23,24], three studies had a score of 7/9 [4,15,25], and two studies of 8/9 [17,26]. Generally, high heterogeneity was observed in study designs, study populations, and oral microbiota evaluation methods.

Of the 12 studies reviewed, 8 assessed the number of *Streptococcus mutans* (SM) and *Lactobacillus* (LB) in saliva [14,15,17,21,24,25,26]. One reported significantly higher levels of SM and LB in saliva in the cleft group compared to the control group [15]. One study found that children with CLP had significantly higher counts of salivary LB, but no significant differences in the SM between the cleft and non-cleft groups were observed [26]. Another study by Sundell et al. [17] found no significant difference in the prevalence of *Lactobacillus* and *Streptococcus mutans*. In Shashni et al. [25] there was no significant difference in the isolation frequency of SM in the cleft group and high-risk caries groups. Three of the included articles focused on evaluating salivary levels of SM and LB in children and adolescents who had been orthodontically treated [14,21,24]. In the study by Parapanisiou et al. [24], 75.6% of the patients in both CLP and non-CLP groups were receiving orthodontic treatment. The authors observed that more than 70% of children from CLP and non-CLP groups had high levels of SM and LB (>10^5^ colony forming units (CFU)/mL). There were no significant differences in the number of SM and LB in saliva between cleft children and the control group. In the article by Antoszewska et al. [21], the study group consisted of patients who were divided into two subgroups according to the presence of cleft palate and the method of treatment (subgroup A1: palatal cleft, malocclusion, fixed appliances; subgroup A2: palatal cleft, malocclusion, removable appliances; subgroup B1: malocclusion, fixed appliances; subgroup B2: malocclusion, removable appliances). The control group consisted of untreated patients with malocclusion and no cleft palate. The study found that, in the group of orthodontically treated children with palatal cleft and malocclusion, percentages of SM and LB levels were lower than in the control group. Additionally, high levels of SM and LB in patients’ saliva were comparably frequent between groups, but statistically significant differences were observed in intergroup comparisons: between A1 and B1 and between A2 and B2. In patients without cleft palate (B1 and B2) a higher percentage of high SM and LB bacteria levels was noted than in the control group. High levels of SM and LB occurred in 75% and 80% of the B1 subgroup and 80% and 85% of the B2 subgroup, respectively. Different results were obtained in patients with cleft palate (group A). High levels of SM and LB were observed in 40% and 45% in subgroup A1 and 45% and 55% in subgroup A2, respectively. In the study by Cheng et al. [14], the control groups comprised patients who were not undergoing orthodontic treatment with fixed appliances. One control group included patients with CLP (cleft control), and the other control group included those without CLP (non-cleft control). The treatment groups included one group of patients with CLP who were undergoing fixed appliance orthodontic treatment (cleft treatment), and the remaining treatment group included patients without CLP who were undergoing fixed appliance orthodontic treatment (non-cleft treatment). The authors observed that patients in the non-cleft treatment group had the highest percentage of patients (86.7%) with ≥10^5^ CFU/mL of MS, while those in the cleft treatment group had the lowest percentage of patients (60%) with ≥10^5^ CFU/mL of MS. For LB, there was a significantly higher percentage of patients with ≥10^5^ CFU/mL of LB in the non-cleft treatment (76.7%) and cleft treatment (73.3%) groups compared to the non-cleft control (46.7%) and cleft control (40.0%) groups. In the study by Lucas et al. [23], plaque was collected from children and matched controls from three different sites, which were (1) the first approximal site distal to the cleft, (2) the contralateral anterior site, and (3) the distal site. The authors observed that there were no differences in the isolation frequency of either SM and LB between the children with CLP and control groups from the matched sites.

Sundell et al. [17] analyzed the salivary microbial profile in children with oral clefts and non-cleft controls in a cross-sectional study using the DNA–DNA hybridization technology. Gram positive *streptococci* (*S. mitis* and *S. gordonii*) dominated salivary profiles in both groups. Among non-*streptococci*, *Fusobacterium nucleatum*, and *Rothia denticariosa* were frequently detected. It is worth noting that both *Lactobacillus* strains were, with a few exceptions, below detection levels. The general observation was that most strains were less frequently detected in the cleft group, and this difference was statistically significant for *Bifidobacterium dentium*, *Fusobacterium nucleatum*, *Streptococcus gordonii*, *Streptococcus mitis*, *Streptococcus salivarius* and *Veillonella parvula*.

The microbial analysis by Perdigokani et al. [7] showed no significant differences in the composition of the subgingival microbiota between the CLP and the control group. Moreover, teeth in the cleft presented higher isolation frequencies and mean percentages of periodontopathic bacteria. The authors found that Gram-negative anaerobic bacteria, some of which are considered putative periodontal pathogens, were present at high isolation frequencies (100%) and high relative proportions (26.2%) in teeth in the cleft compared to corresponding teeth in the control group. *Fusobacterium* spp. were common in children and adolescents with cleft, at higher proportions compared to their non-cleft peers. Another analysis of subgingival microorganisms showed that *Prevotella nigrescens* was detected in 16.67% of the cleft group and 11.11% of the control group, while *Porphyromonas gingivalis* and *Treponema denticola* were not detected [16]. One study looked at dental plaque microbiota in CLP patients using metatranscriptomic analysis [22]. The composition of bacteria between the groups was not clearly different, and the 30 most important genera were similar in all groups. *Actinomyces* was the most dominant genus in both groups, with *Corynebacterium matruchotii* and *Leptotrichia hofstadii* at the species level. The authors concluded that bacterial composition and functional profiles alone did not provide significant signs of dysbiosis in the CLP group. However, group-specific active taxa were identified, including *Streptococcus* and *Prevotella* species in the CLP group. *Prevotella* was the most commonly detected genus in the plaque-biofilms collected from teeth located within the cleft and teeth adjacent to the cleft site of individuals with CLP compared to those obtained from the teeth of healthy patients. Specifically, *P. marshii*, *P. micans*, *P. nigrescens*, *P. pallens*, and *P. pleuritidis* have been reported to be more commonly associated with CLP, while *P. melaninogenica* and *P. oralis* have been found to be more prevalent in non-CLP individuals [7,22]. Regarding *Streptococcus*, *S. anginosus*, *S. cristatus*, *S. gordonii*, and *S. salivarius* were found to be associated with CLP, while *S. oralis* and *S. sanguinis* were more commonly detected in the control group [22]. However, there was no significant difference in the prevalence of *S. intermedius* and other *Streptococcus* species between the cleft and non-cleft groups. Among *Lactobacillus* species, *L. fermentum*, *L. rhamnosum*, and *L. vaginalis* were found to be more prevalent in individuals with CLP [7,22]. Moreover, the network structure differed between groups; *Actinomyces johnsonii* and several species in the CLP group were active taxa that were linked based on positive correlations with statistical significance [22].

One study focused on identifying *Candida albicans* and other *Candida* species in children and adolescents with clefts and healthy controls in three age groups [4]. The authors found that the *Candida* colonization rate in patients with cleft (63.3%) was significantly higher than in healthy control patients (18.3%). *Candida* colonization rate and *C. albicans* differed by age but was not significantly related to gender in cleft patients and healthy controls. The rate of asymptomatic carriage of *Candida* species in the oral cavity in patients with cleft was 63.3%, where *C. albicans* was the most commonly detected species, followed by *C. glabrata*. *C. kefyr* was the least frequently detected species. In contrast, *Candida* species were isolated from 18.3% control patients, and only two species were detected, namely *C. albicans* and *C. kefyr*.

Clinical parameters of the teeth and periodontium were also examined in all included studies. The results of the clinical examination are shown in Table 2. Most studies showed that patients with cleft had significantly worse oral hygiene [4,7,15,16,21,24,25] and higher dmft/DMFT (decayed, missing, filled deciduous/permanent teeth) scores than healthy control groups [4,17]. In contrast, one study found no significant differences between CLP and control groups in clinical parameters, such as gingival index (GI), oral hygiene index (OHI), and dmft/DMFT scores [23]; two showed no significant differences for dmft/DMFT scores [24,25] between patients with and without cleft. One study found that oral hygiene did not differ between groups [17]. Cheng et al. [14] found the lowest mean DMFT in the cleft group.

## 4. Discussion

The human oral cavity provides a habitat for oral microbial communities. The complexity of its anatomical structure and its connectivity to the external and moist environment contribute to the complexity and ecological specificity of the microbiome colonized in it. Complex endogenous and exogenous factors affect the occurrence and development of the oral microbiota and maintain it in a dynamic balance. The dysbiosis of the oral microbiome, when the microecological balance between host and microorganisms is disturbed, can induce of oral infectious diseases, such as caries, oral cancer, oral candidiasis, and periodontal disease, and reflects oral and general health status [35,36].

This systematic review presents a comprehensive analysis of the oral microbiota in children with CLP. In light of the increased prevalence of dental caries in these children [37], it is essential to analyze how the oral microbiota of children with CLP differs from the microbiological status of the healthy population without cleft, as well as which bacterial species, particularly those associated with caries, may be associated with the plaque biofilms formed on the teeth of these patients [38]. Twelve studies investigating the microbiological status of CLP individuals compared to non-cleft individuals were included in the review. As the overall findings showed higher incidence of caries in in children with CLP compared to those without [37], the majority of the included studies concentrate primarily on the assessment of caries-associated microorganisms, such as SM and/or LB [14,15,17,21,24,25,26]. Only two articles focused on the evaluation of the composition of the subgingival microbiota in children with CLP [7,16]. The results obtained in these studies exhibit variations. Some studies reported significantly higher levels of SM and/or LB in the saliva of the cleft group compared to the control group [15,26]. However, other studies failed to confirm such differences when comparing with non-cleft controls [14,17,21,23,24]. Importantly, these studies highlighted the impact of fixed and removable orthodontic appliances on the colonization of cariogenic SM and the late colonizer LB. This colonization phenomenon is particularly noteworthy as it poses a negative influence on caries-susceptible patients with CLP undergoing orthodontic treatment, making it more challenging to maintain good oral hygiene practices [14,21]. Similarly, studies focusing on subgingival plaque microbiota in young patients with clefts did not show significant differences between cleft and non-cleft patients [7,16]. When analyzing these results, it is important to consider that individuals with clefts underwent surgical intervention to correct clefts within the first year of life, according to the recommended procedure worldwide. This early intervention results in the normalization of anatomical structures and feeding practices, but also may enable a normal, non-compromised colonization of the oral biofilm in individuals with clefts. However, the authors emphasized that, although there may not be significant differences in the subgingival microbiota of children with CLP when compared to matched children without clefts, there is a noticeable tendency for increased isolation frequencies and relative proportions of putative periodontal pathogens in young children with clefts. This suggests that, even though the differences may not be major, there is still a discernible trend towards a higher presence of potentially harmful microorganisms in the oral microbiota of young children with clefts [7]. On the other hand, a study utilizing metatranscriptomic analysis revealed a significant functional dysbiosis within the plaque microbiota of individuals with CLP when compared to the control group [22]. This dysbiosis was characterized by differences in the network structure, potentially linked to increased susceptibility to dental caries. However, it is important to note that this functional dysbiosis may not be visually evident within the oral microbiota of CLP patients. It was emphasized that, by considering factors beyond mere appearance, we can gain a deeper understanding of the complex interactions between microbial colonization, oral health, and the unique challenges faced by individuals with CLP. Furthermore, in light of these different findings, the investigators demonstrated that the composition of oral microflora depends individually on the sample site, current health, or dental status. In addition to technical variations in sample collection and processing, it is crucial to take into account the potential influence of various factors, such as patients’ hygiene and dietary practices, cultural differences, and genetic variations, that can lead to different colonization patterns of the oral microbiota in different regions of the world [39,40,41]. These factors can contribute to the diversity of the oral microbiota, further emphasizing the need for comprehensive consideration of them in research.

In this context, when interpreting the results of this systematic review, we should be aware that the included studies show differences in sample selection, study designs, and oral microbiota detection methods used. It should be noted that there are some limitations related to the small size [7,16,22,24] and the large age-range of the study samples [7,15,21,22,23,24]. On the other hand, in the study by Sundell et al. [26] the number of participants was considerable. It is also important to take into consideration some patients’ characteristics, such as gender or type of cleft. In this aspect, it should be noted that one of the studies assessed oral microbiota with respect to factors such as age, gender, surgical treatment, type of cleft, and oral health [4]. In three articles, the cleft group comprised children with different types of clefts; however, the authors did not analyze the results in relation to the cleft subgroups [15,22,24]. As mentioned earlier, another factor that may affect oral microbiota is the use of the orthodontic appliances. Some of the reviewed studies analyzed salivary levels of SM and LB considering the presence or absence of the orthodontic treatment [14,21], as well as the type of orthodontic appliances [21]. Another two studies compared cleft and non-cleft children matched for age, gender, and orthodontic treatment [7,24]. Patients orthodontically treated with fixed or removable appliances also participated in the study by Funahashi et al. [22]. However, in that study, the number of participants was very small, and the authors did not analyze the impact of orthodontic treatment on the study results.

It is important to emphasize that this systematic review includes papers from 2000 [23] to 2019 [22], and progress in microbiology techniques over these two decades from culture to more advanced culture-independent gene techniques could have significant impact on the results. The cultivation of oral bacterial species requires distinctive conditions, such as biochemically defined media, an anaerobic environment, proper incubation temperature, and diverse PH. However, not all bacteria can be cultivated in the laboratory [42,43]. The currently developed culture-independent approaches enable us to identify, classify, and characterize the uncultivable microbiological genera [44]. To date, almost 50% of oral microbial species detected through culture-independent gene sequencing techniques remain resistant to cultivation [43]. In this systematic review, most of the abovementioned studies used cultivation [4,7,15,23] and simple chair-side techniques [14,21,24,25,26] for the microbial evaluation, which provided limited information on the broader microbial profile. One paper [17] used more advanced methods, such as the checkerboard DNA–DNA hybridization technology with 12 pre-determined bacterial probes to analyze saliva samples. This method is considered to be useful for the enumeration of bacterial species in complex microbial systems, such as the oral biofilm. However, the shortcomings of this method are possible cross-reactions and varying reproducibility for different strains [45]. Furthermore, the authors emphasized that the probes used were not entirely specific enough to differentiate between genotypes of the same species, meaning that the selected *Lactobacillus* strains may not have been present in sufficient amounts to be detected. They also noted that the present assay only mirrored 12 selected strains out of the over 600 prevalent taxa at species level that are reported from the oral cavity [17]. On the other hand, Funahashi et al. [22] used metatranscriptomic analysis to detect oral microorganisms through comprehensive analysis of bacterial mRNA abundance. This analysis captures the transcriptional activity of every bacterial species in the microbiota. Metatranscriptomics holds great potential to uncover biological information that may be obscured by other genomic methodologies [46]. In summary, it should be noted that the use of the current molecular-based detection approaches has expanded our awareness of the diversity, architecture, and function of oral microorganisms. In light of the above, when interpreting the results of the studies on oral microbiota in cleft patients, the variety of microbiological methods used to identify microorganisms should be considered, along with their advantages and shortcomings. Further research that includes a larger number of patients and more homogenous samples and takes into consideration different factors, e.g., gender or orthodontic treatment. Using more advanced technologies to detect oral bacteria is necessary to deepen our understanding of the oral microbiota and potential implications for oral health management in CLP children.

Yeast-derived fungi, which include all of the *Candida* species, have not been clearly classified as strictly pathogenic. Their importance in the oral biocenosis has not yet been precisely explained. Some authors believe that they are present in the healthy oral cavity as commensal microorganisms, and thus are part of its physiological microflora. On the other hand, according to other researchers, *Candida* fungi are pathogens as they are the cause of systemic mycoses of many organs, including the oral cavity [47,48]. *Candida albicans* is particularly pathogenic and has a high capacity to form biofilms [35,49,50,51]. One study in this review which focused on the prevalence of *Candida* species showed that the colonization rate of *Candida* in patients with cleft was significantly higher than in healthy controls [4]. Moreover, the presence of specific cleft types and the number of surgical interventions were correlated with an elevated colonization of *Candida*. This heightened *Candida* colonization in CLP patients could be attributed to a compromised protective function of the oral mucosa due to tissue deficiencies, as well as factors related to surgeries and hospital environment. Additionally, the prevalence of *Candida* species and the distribution of *Candida albicans* varied across different age groups among patients with cleft and healthy controls, likely influenced by physiological, environmental, and dental-related factors. Furthermore, it was underscored that individuals with cleft displayed poorer oral health compared to unaffected children. Hence, a particular focus should be directed towards monitoring the oral health status of CLP patients and implementing preventive measures to avoid the development of oral candidiasis, especially in cases requiring multiple surgical procedures [4].

From a clinical perspective, most studies analyzing the oral microbiota showed that patients with cleft had a significantly poorer oral hygiene [4,7,15,16,21,24,25]. This can be attributed to several factors. Firstly, the presence of residual scar tissue resulting from multiple surgical procedures in the cleft region can make tooth cleaning challenging. Moreover, individuals with CLP often have a range of other health issues, such as otitis media, speech difficulties, and a common fear of brushing their teeth in the cleft area. These factors can lead to a lack of interest or motivation in maintaining proper oral hygiene. Additionally, the presence of malocclusions and structural abnormalities, including changes in tooth number, position, and morphology, further complicate oral hygiene maintenance. These issues are often accompanied by long orthodontic treatments, which can prolong the challenges faced by these patients in maintaining good oral hygiene [12,14,15,26,38,52]. Furthermore, fixed orthodontic appliances, such as bands and braces, pose additional challenges to oral hygiene. These components can create areas where plaque accumulation is more likely, making thorough cleaning more difficult. Studies have also highlighted the impact of these fixed appliances on oral hygiene in individuals with CLP [39,53]. Overall, the combination of factors, including the cleft deformity, collapse of the maxillary segments, orthodontic anomalies, scarring, and inelastic upper lip resulting from corrective surgeries, contribute to the difficulties in achieving proper oral hygiene in individuals with CLP [54]. Given these challenges, it becomes crucial to implement individualized preventive oral health programs specifically tailored to the needs of CLP patients. Recognizing and addressing these factors is imperative to improve oral hygiene and overall oral health outcomes in this population [7]. On the other hand, the findings of the studies focused on the dmft/DMFT scores in cleft children are not so clear. Some studies found higher dmft/DMFT scores that in healthy controls [4,17], however others showed no differences between the CLP and control groups in the dmft/DMFT indices [23,24,25]. When analyzing these studies results, it is important to note that the existing literature may present inconsistent results, which can be attributed to the multifaceted nature of dental caries, variations in research methodologies, disparities in awareness regarding the significance of excellent oral health, diverse dietary habits, and potential racial differences [14,22]. Furthermore, the provision of oral health care and orthodontic treatment to address dentofacial deformities associated with CLP can vary significantly worldwide. This variation in care and treatment approaches may also contribute to inconsistencies in research outcomes.

Given these considerations, it becomes even more imperative to prioritize and deliver consistent, comprehensive oral health care to individuals with CLP. This includes educating patients and caregivers about the importance of excellent oral hygiene practices, promoting awareness of dietary habits that support oral health, and implementing individualized preventive strategies. It is crucial to prioritize primary preventive strategies for oral health in children with CLP from an early age, particularly those at high risk of dental caries and those undergoing orthodontic treatment. Implementing consistent oral hygiene practices, such as brushing thoroughly three times a day, using fluoridated toothpaste, incorporating irrigators or mouthwashes, and limiting the consumption of sugary foods and drinks, can significantly contribute to maintaining oral health in this population. Emphasizing the importance of primary preventive strategies, integrating pediatric dental services, and implementing standardized protocols in cleft centers worldwide will empower these children to maintain optimal oral health.

This systematic review presents some limitations that should be acknowledged: (a) high heterogeneity was observed in study designs, study populations, and oral microbiota evaluation methods of the included research; (b) the majority of the included studies concentrate primarily on the assessment of caries-associated microorganisms, such as SM and/or LB; (c) this review includes papers from 2000 to 2019 and progress in microbiology techniques in this time could have impact on the results; (d) many other factors, such as the sample site, technical variations in sample collection and processing, as well as patients’ current health status, their hygiene and feeding practices or genetic variations, may affect the results among the included studies; (e) we should also be aware that the differences in the study groups’ characteristics, such as gender, age, and type of cleft, may affect the results; (f) as many factors can contribute to the diversity of the oral microbiota in cleft children, future studies are needed.

## 5. Conclusions

In summary, as the results of the aforementioned studies are not conclusive, further research focused on the oral microbiota in CLP children are needed. Furthermore, it is important to note that many detected species have deleterious effects on specific tissues or are associated with serious diseases, including oral infectious diseases, and this makes it imperative to definitively investigate the microbiota of CLP patients and have a more complete understanding of their condition. It is crucial to establish a standardized approach to oral health care and orthodontic treatment, taking into account the unique needs and challenges associated with CLP.

## Figures and Tables

**Table 1:** Characteristics of the study included.

Authors, Year	Study Groups, Participants	Study Design	Methods Used to Detect Oral Microbiota
Ahluwalia et al., 2004 [15]	Children aged 6–16 years, with (*n* = 81) or without (*n* = 61) cleft palates, were studied. The cleft group included patients with unilateral cleft lip (*n* = 6), unilateral cleft and alveolus (*n* = 9), bilateral cleft and alveolus (*n* = 5), UCLP (*n* = 29), BCLP (*n* = 16), CP only (*n* = 11), soft palate (*n* = 3), and other cleft condition (*n* = 2).	Caries status was determined according to WHO criteria supplemented by radiographs. Oral health status was assessed as oral hygiene index, gingival index, and plaque index. Approximately 2 mL of unstimulated mixed saliva was collected from each child. The number of SM, LB, and *Candida* in each saliva sample were determined.	Saliva samples were decimally diluted in FAB (IPT Ltd., Bury, Lancs, UK), and a 100-µL quantity of appropriate dilutions was spread-plated onto selective media for yeasts (SAB; Oxoid, Basingstoke, Hants, UK), LB (ROG; Oxoid, Basingstoke, UK), SM and *Streptococcus sobrinus* (BMSA; Becton Dickinson, Cowley, Oxon, UK).
Antoszewska et al., 2010 [21]	The study group comprised 200 patients aged 6–21 years chosen consecutively, based on the type of orthodontic approach (fixed or removable appliances) and CP presence. The control group (50 individuals) consisted of untreated patients with malocclusion and no cleft palate.	Oral hygiene state was described by the PI and GI indexes. Saliva samples were collected to determine the presence of SM and LB. In total, saliva was collected from 100 patients.	The CRT bacteria test (Vivadent company, Vienna, Austria) was used to evaluate the quantity of salivary MS and LB. The CFU of bacteria SM and LB were observed and visual assessed. The salivary level of SM and LB was evaluated as high in the case of CFU/mL ≥ 10^5^ or low when CFU/mL < 10^5^.
Cheng et al., 2007 [14]	One hundred and ten patients aged between 12 and 17 years were recruited into one of four different groups, comprising two control groups and two treatment groups. The control groups consisted of patients with and without CLP who were not undergoing orthodontic treatment. The treatment groups comprised of patients with and without CLP undergoing orthodontic treatment.	The total number of DMFT was calculated and recorded. The salivary secretion time, pH of resting and stimulated saliva, salivary flow rate, buffering capacity, quantity of MS and LB in saliva were measured.	The CRT bacteria^®^ (Vivadent Ets., Vienna, Austria) test was used to evaluate the quantity of salivary MS and LB. The CFUs of salivary SM and LB were assessed and categorized to either a low-density category (<10^5^ CFU/mL) or to a high-density category (≥10^5^ CFU/mL).
Costa et al., 2003 [16]	Fifty-seven selected children, including 30 with UCLP (experimental group) and 27 without clefts (control group) between the ages of 5 and 6 years.	During clinical examination PI and GI indexes were determined. Four samples of subgingival plaque were collected from each child in the control group. In the experimental group, two additional samples were collected.	Evaluation of bacteria, having standard antigens from *Porphyromonas gingivalis* (ATCC 33277, American Type Culture Collection, Manassas, VA, USA), *Prevotella nigrescens* (ATCC 33563), and *Treponema denticola* (ATCC 35405), and the determination of carbohydrate amount present in plaque samples were accomplished through the slot immunoblot (SIB) assay.
Funahashi et al., 2019 [22]	CLP (*n* = 6) and non-CLP (*n* = 4) patients undergoing orthodontic treatment aged 7–15 years old. All CLP patients were treated with plastic surgery of the lip and palate in childhood.	Clinical parameters: OHI, GI, DMFT for deciduous and permanent teeth. Microbiological parameters: total bacterial RNA was extracted from each sample and sequenced.	Metatranscriptomic analysis: Bacterial composition and functional profiles were estimated from 16S rRNA and mRNA reads, respectively. Species listed in both rRNA-based and mRNA-based bacterial composition were identified as viable taxa with in situ function (VTiF), and the VTiF with a high mRNA-to-rRNA ratio were considered to be transcriptionally active.
Lucas et al., 2000 [23]	Sixty children with UCLP aged between 3 and 15 years and matched controls. The mean age of the patients was 9.1 ± 3.1 years and of the control group 8.8 ± 3.2 years. There were 36 boys and 24 girls in each group.	The DMFT and DMFS scores in both deciduous and permanent dentition were calculated. The plaque and gingivitis scores were also recorded. Plaque was collected from 25 of the children and matched controls from three different sites: (1) the first approximal site distal to the cleft, (2) the contralateral anterior site and (3) the distal site. It was cultured for SM and LB. Plaque was collected from two sites in matched control groups.	One hundred µL aliquots of the appropriate dilutions were inoculated onto both selective and nonselective Media for SM (BMSA) and LB (Rogosa agar, Oxoid Unipath, Basingstoke, UK). Columbia agar supplemented with 5% (*v*/*v*) defibrinated horse blood (CBA) was prepared to determine the total aerobic and anaerobic counts.
Parapanisiou et al., 2009 [24]	The CLP group consisted of 41 children and adolescents (23 boys and 18 girls, 4–18 years-old; mean age 10.54 ± 3.37 years) with UCLP (*n* = 26), BCLP (*n* = 10) and CP (*n* = 5); 75.6% of patients were under orthodontic treatment. The control group consisted of 41 healthy children and adolescents with mean age of 10.7 ± 3.03 years, matched for age, gender and orthodontic treatment.	Oral hygiene was assessed using PI. The prevalence of initial/white spots and cavitated carious lesions, as well as hypoplasia was evaluated. Stimulated saliva was collected after chewing paraffin gum for 5 min.	The number of LB and MS in saliva was detected using the CRT^®^ chairside tests (Ivoclar-Vivadent, Vienna, Austria).
Perdikogianni et al., 2009 [7]	Forty-one children and adolescents aged 4–18 years with CLP and 41 healthy controls participated in the study.	The clinical parameters examined were the PI, GI and CPITN. Samples of subgingival plaque were collected from 21 randomly selected patients from each group.	For morphological evaluation of the bacterial cells dark field microscopy was used. Aliquots of 0.1 mL of the appropriate dilutions were plated in duplicate onto ETSA supplemented with 4% defibrinated human blood to determine the composition of the predominant cultivable microbiota and onto TSBV (BBL Microbiology systems, Cockeysville, MD, USA) to isolate *Aggregatibacter actinomycetemcomitans*.
Rawashdeh et al., 2011 [4]	Sixty patients with cleft: UCLP, BCLP, and CP and 60 control patients aged 10.1 ± 6.71 and 9.75 ± 7.26 years. Patients with cleft and healthy controls were divided into three age groups: those aged 5 years and younger (age group 1), those aged 6 to 16 years (age group 2), and those who were aged 17 years and older (age group 3).	Oral health status was assessed using the GI and the PI. Dental caries was assessed by recording the DMFT/dmft index. A culture swab was obtained from the tongue and buccal and palatal mucosae.	*Candida albicans* and other *Candida* species were identified using the germ tube test and the commercially available biochemical test panel VITEK (bioMérieux, Marcy l’Etoile, France) and the Yeast Biochemical Card Pinsert (bioMérieux).
Shashni et al., 2015 [25]	Seventy-three children in the age range of 4–9 years comprised three groups: Group I (*n* = 23, children with CLP), Group II (*n* = 25, non-cleft high caries risk children) and Group III (*n* = 25, non-cleft caries free children). All children included in the three study groups were matched for age, sex and social class. The mean age of children in the Group I, Group II and Group III were 6.55 ± 1.8 years, 6.09 ± 1.3 years and 7.01 ± 1.2 years, respectively.	Various risk factors for dental caries like type of oral hygiene practice, sugar exposures/day, developmental defects of enamel, caries activity, salivary SM and LB levels were evaluated.	The levels of SM in saliva of the children were analyzed using Dentocult SM “Strip Mutans Test Kits” (Orion Diagnostica, Helsinki, Finland). *Lactobacilli* levels were assessed by conventional method using Mann Rogosa Sharpe agar.
Sundell et al., 2015 [26]	The study group consisted of 133 children with CLP (77 patients aged 5 years and 56 aged 10 years) and 297 non-cleft controls (133 aged 5 years and 164 aged 10 years).	Oral hygiene was assessed using the QH plaque index. Caries prevalence and frequency were scored according to the ICDAS. Whole saliva samples were analysed for SM, LB, buffering capacity and secretion rate.	Buffer capacity (Dentobuff^®^ Strip, Espoo, Finland), SM (Dentocult^®^ SM-Strip mutans, Espoo, Finland) and LB (Dentocult^®^ LB, Espoo, Finland) counts were estimated with commercial chair-side tests purchased from Orion Diagnostica, Espoo, Finland.
Sundell et al., 2018 [17]	The cleft group consisted of 80 children aged 5 years, and 144 age-matched non-cleft children were recruited as a control group.	Gingival inflammation was evaluated as “yes” or “no” after bleeding during gentle probing. Dental plaque was scored according to the modified QH index. Caries was scored according to the ICDAS. Stimulated whole saliva samples were collected.	The samples were processed with the checkerboard DNA–DNA hybridization technology using 12 pre-determined bacterial probes according to the department collection, Oral Microbiology, Gothenburg, Sweden (OMGS): *Actinomyces naeslundi* 2466; *Actinomyces odontolyticus* G67; *Bifidobacterium dentium* G174; *Fusobacterium nucleatum* 2865; *Lactobacillus casei* 3184; *Lactobacillus salivarius* 3830; *Rothia denticariosa* 1956; *Streptococcus gordonii* 2471; *Streptococcus mitis* 1770; *Streptococcus mutans* 2482; *Streptococcus salivarius* 2473; *Veillonella parvula* G186.

CLP—cleft lip and palate; UCLP—unilateral cleft lip and palate; BCLP—bilateral cleft lip and palate; CP—cleft palate; FAB—Fastidious Anaerobe Broth; SAB—Sabouraud Dextrose Agar; ROG—Rogosa Agar; BMSA—Mitis Salivarius Agar supplemented with sucrose and bacitracin; CFU—colony-forming units; ETSA—Enriched Trypticase Soy Agar; TSBV—trypticase-soy-agar supplemented with 5% serum 75 lg ⁄mL bacitracin and 5 lg⁄mL vancomycin; CPITN—Community Periodontal Index of Treatment Needs [27]; dmft (deft)/DMFT—decayed, missing (extracted), filled deciduous and permanent teeth [28]; DMFS—decayed, missing and filled permanent surfaces; GI—gingival index [29,30]; ICDAS—International Caries Detection and Assessment System [31]; OHI—oral hygiene index [32]; PI—plaque index [33]; QH index—Quigley–Hein plaque index [34]; SM—*Streptococcus mutans*; LB—*Lactobacillus*; VITEK—biochemical test panel [4].

**Table 2:** The clinical and microbiological results of the included studies.

Authors, Year	Results
Ahluwalia et al., 2004 [15]	Clinical: Children with cleft palates had DMFT and dmft scores greater than those of the control group. The oral hygiene, plaque, and gingival index scores were greater in the CP group. Microbiological: The salivary levels of SM, LB, and Yeasts were significantly greater in the cleft palate children than in the control group.
Antoszewska et al., 2010 [21]	Clinical: The distribution of the plaque index values in patients with CP was different from that in patients without cleft, and the observed intergroup difference was statistically significant. A statistically significant correlation of high plaque index values with an increase in SM and LB colonization was observed. GI values did not statistically significantly differentiate patients. Microbiological: The study found that in the group of orthodontically treated children with palatal cleft and malocclusion, percentages of SM and LB levels were lower than in the control group.
Cheng et al., 2008 [14]	Clinical: The average DMFT of the cleft treatment group was the lowest among all groups. Microbiological: There was a significant difference in the percentage of patients with ≥10^5^ CFU/mL of MS between the cleft treatment and non-cleft treatment groups. Patients in the non-cleft treatment group had the highest percentage of patients (86.7%) with ≥10^5^ CFU/mL of MS, while patients in the cleft treatment group had the lowest percentage of patients (60%) with ≥10^5^ CFU/mL of MS. For LB, there was significantly higher percentage of patients with ≥10^5^ CFU/mL of LB in the non-cleft treatment (76.7%) and cleft treatment (73.3%) groups compared to the non-cleft control (46.7%) and cleft control (40.0%) groups.
Costa et al., 2003 [16]	Clinical: The mean PI in the CLP group was higher than in the control group, although this difference was not statistically significant. The mean GI in the CLP group was significantly higher than in the control group. Microbiological: Analysis of the organisms showed that *Prevotella nigrescens* was detected in 16.67% of the experimental group and 11.11% of the control group, while *Porphyromonas gingivalis* and *Treponema denticola* were not detected.
Funahashi et al., 2019 [22]	Clinical: No significant differences were observed between the CLP and control groups in terms of age and clinical parameters. Microbiological: Bacterial composition between groups was not clearly different, and the 30 most important genera were similar between groups. *Actinomyces* was the most dominant genus in both groups (on average 14.0% in the CLP group and 10.6% in the control group). At the species level, *Corynebacterium matruchotii* and *Leptotrichia hofstadii* were the most predominant in both groups. A group-specific active taxa were identified, including *Streptococcus* and *Prevotella* species in the CLP group.
Lucas et al., 2000 [23]	Clinical: There was no significant difference in the caries, plaque and gingivitis scores between children with cleft palate and the controls. Microbiological: There was no significant difference in the proportion of SM or LB at the cleft site, compared to the unaffected site in the study group, although there was an anterior-posterior gradient in the proportion of *S. mutans*. There was no significant association between the stagnation area at the cleft site and the bacteria associated with dental caries.
Parapanisiou et al., 2009 [24]	Clinical: PI of total dentition was significantly higher in the CLP than in the control group and the same applied to the teeth adjacent to the cleft. CLP patients undergoing orthodontic treatment had worse oral hygiene than those in the non-cleft group. No significant differences were found in the dmft/DMFT scores between patients with cleft and without cleft. Microbiological: More than 70% of children in both CLP and control groups had high levels of SM and LB (>10^5^ CFU/mL). Levels of SM and LB as well as the quality of the saliva were similar in both groups. Patients in both CLP and non-cleft group under orthodontic treatment had high levels of cariogenic bacteria.
Perdikogianni et al., 2009 [7]	Clinical: The CLP group had significantly higher PI rate compared to the control group. Children in both groups presented a mild degree of gingivitis. Microbiological: Microbial analysis did not reveal significant differences in the composition of the subgingival microbiota between the groups. Teeth in the cleft presented a higher isolation frequency and mean percentage of periodontopathic bacteria.
Rawashdeh et al., 2011 [4]	Clinical: Patients with cleft had significantly worse health status than healthy controls; however, this was not influenced by the type of cleft or the number of surgeries. Patients with cleft showed significantly higher GI and dmft/DMFT scores than the control group. Similarly, PI in patients with cleft was higher than controls, but the difference was not statistically significant. Microbiological: The colonization rate of *Candida* in patients with cleft (63.3%) was significantly higher than in healthy control patients (18.3%). The candidal colonization rate was highest in patients with cleft who had at least three surgeries (78.2%) and in patients with bilateral clefts (77.7%). The rate of asymptomatic oral carriage rate of *Candida* species in patients with cleft was 63.3%, with *C. albicans* being the most frequently detected species (81.5%), followed by *C. glabrata* (10.5%).
Shashni et al., 2015 [25]	Clinical: The mean deft score among Group II children was significantly higher as compared to the Group I children. The mean deft + DMFT score among Group I and Group II children was comparable. Microbiological: The salivary SM levels in Group I and Group II children were higher when compared to LB counts. There was no significant difference in the isolation frequency of SM in the cleft group and high caries risk group. The levels of SM > 10^6^ CFU/mL (Score 3) were found in 60% of Group II children, 21.7% of Group I children, and only in one child in the caries free group (Group III). There was a statistically significant difference between Group I and Group III children and between Group I and Group II children.
Sundell et al., 2015 [26]	Clinical: Children with clefts displayed poorer oral hygiene. Microbiological: Children with CLP had significantly higher counts of salivary *lactobacilli*.
Sundell et al., 2018 [17]	Clinical: Children in the cleft group had a significantly higher prevalence of dental caries. Oral hygiene did not differ between the groups. Microbiological: Overall, children with cleft displayed a significantly lower prevalence of common commensal species (e.g., *Streptococcus, Fusobacterium*) compared to the control group without cleft.

CLP—cleft lip and palate; CP—cleft palate; CFU—colony-forming units; dmft (deft)/DMFT—decayed, missing (extracted), filled deciduous and permanent teeth [28]; GI—gingival index [29,30]; PI—plaque index [33]; SM—*Streptococcus mutans*; LB—*Lactobacillus.*

**Table 3:** The quality assessment of the studies included (NOS assessment).

Authors, Year	Selection	Comparability	Outcome	Total Score
Ahluwalia et al., 2004 [15]	***	**	**	7
Antoszewska et al., 2010 [21]	**	**	**	6
Cheng et al., 2007 [14]	**	**	**	6
Costa et al., 2003 [16]	**	**	**	6
Funahashi et al., 2019 [22]	**	**	**	6
Lucas et al., 2000 [23]	**	**	**	6
Parapanisiou et al., 2009 [24]	**	**	**	6
Perdikogianni et al., 2009 [7]	**	**	**	6
Rawashdeh et al., 2011 [4]	***	**	**	7
Shashni et al., 2008 [25]	***	**	**	7
Sundell et al., 2015 [26]	****	**	**	8
Sundell et al., 2018 [17]	****	**	**	8

A study can be awarded a maximum of one star for each numbered item within the selection and outcome catego-ries. A maximum of two stars can be given for comparability. * are assigned to three criteria, i.e., selection (with a maximum of 4 stars [****]), comparability (with a maximum of 2 stars [**]), and outcome (with a maximum of 3 stars [***]).

## Data Availability

All data are available in the studies included in the review and were discussed in the present manuscript.

## Supplementary Materials

The following supporting information can be downloaded at: https://www.mdpi.com/article/10.3390/jcm12185867/s1; Table S1: PRISMA 2020 abstract checklist; Table S2: PRISMA 2020 manuscript checklist.
